# Dibromido[*N*-propyl-*N*′-(2-pyridylmethyl­idene)ethane-1,2-diamine]zinc(II)

**DOI:** 10.1107/S1600536808023660

**Published:** 2008-07-31

**Authors:** Xue-Wen Zhu, Xu-Zhao Yang

**Affiliations:** a Key Laboratory of Surface and Interface Science of Henan, School of Materials and Chemical Engineering, Zhengzhou University of Light Industry, Zhengzhou 450002, People’s Republic of China

## Abstract

The title complex, [ZnBr_2_(C_11_H_17_N_3_)], is a mononuclear zinc(II) compound derived from the Schiff base *N*-propyl-*N*′-(1-pyridin-2-ylmethyl­idene)ethane-1,2-diamine. The Zn^II^ atom is five-coordinate, binding to the imine N, pyridine N, and amine N atoms of the Schiff base ligand and to two bromide anions in a distorted trigonal-bipyramidal coordination geometry. Adjacent mol­ecules are linked through inter­molecular N—H⋯Br hydrogen bonds, forming dimers.

## Related literature

For background to the chemistry of Schiff base complexes, see: Ali *et al.* (2008 ▶); Biswas *et al.* (2008 ▶); Chen *et al.* (2008 ▶); Darensbourg & Frantz (2007 ▶); Habibi *et al.* (2007 ▶); Kawamoto *et al.* (2008 ▶); Lipscomb & Sträter (1996 ▶); Tomat *et al.* (2007 ▶); Wu *et al.* (2008 ▶); Yuan *et al.* (2007 ▶). For related structures, see: Dapporto *et al.* (2001 ▶); You & Zhu (2006 ▶).
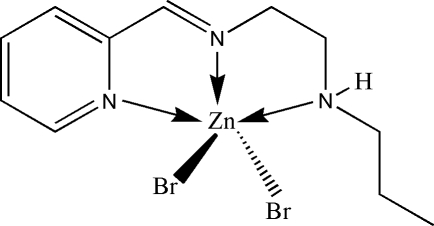


## Experimental

### 

#### Crystal data

[ZnBr_2_(C_11_H_17_N_3_)]
*M* *_r_* = 416.47
Monoclinic, 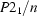

*a* = 8.252 (4) Å
*b* = 12.249 (5) Å
*c* = 14.726 (6) Å
β = 94.562 (7)°
*V* = 1483.8 (11) Å^3^
*Z* = 4
Mo *K*α radiation
μ = 7.02 mm^−1^
*T* = 298 (2) K
0.32 × 0.30 × 0.30 mm

#### Data collection

Bruker APEXII CCD area-detector diffractometer
Absorption correction: multi-scan (*SADABS*; Sheldrick, 2004 ▶) *T*_min_ = 0.212, *T*_max_ = 0.227 (expected range = 0.114–0.122)
12333 measured reflections
3378 independent reflections
2167 reflections with *I* > 2σ(*I*)
*R*_int_ = 0.083

#### Refinement

*R*[*F* ^2^ > 2σ(*F* ^2^)] = 0.045
*wR*(*F* ^2^) = 0.105
*S* = 1.01
3378 reflections
159 parameters
1 restraint
H atoms treated by a mixture of independent and constrained refinement
Δρ_max_ = 0.91 e Å^−3^
Δρ_min_ = −0.80 e Å^−3^

### 

Data collection: *APEX2* (Bruker, 2004 ▶); cell refinement: *SAINT* (Bruker, 2004 ▶); data reduction: *SAINT*; program(s) used to solve structure: *SHELXS97* (Sheldrick, 2008 ▶); program(s) used to refine structure: *SHELXL97* (Sheldrick, 2008 ▶); molecular graphics: *SHELXTL* (Sheldrick, 2008 ▶); software used to prepare material for publication: *SHELXTL*.

## Supplementary Material

Crystal structure: contains datablocks global, I. DOI: 10.1107/S1600536808023660/sj2523sup1.cif

Structure factors: contains datablocks I. DOI: 10.1107/S1600536808023660/sj2523Isup2.hkl

Additional supplementary materials:  crystallographic information; 3D view; checkCIF report

## Figures and Tables

**Table:** 

Zn1—N2	2.095 (4)
Zn1—N3	2.202 (4)
Zn1—N1	2.303 (4)
Zn1—Br2	2.3954 (13)
Zn1—Br1	2.4102 (11)

**Table:** 

N2—Zn1—N3	77.83 (16)
N2—Zn1—N1	73.04 (15)
N3—Zn1—N1	149.43 (15)
N2—Zn1—Br2	131.24 (11)
N3—Zn1—Br2	100.86 (11)
N1—Zn1—Br2	91.57 (11)
N2—Zn1—Br1	111.33 (12)
N3—Zn1—Br1	99.08 (11)
N1—Zn1—Br1	99.94 (11)
Br2—Zn1—Br1	116.88 (4)

**Table 2:** Hydrogen-bond geometry (Å, °)

*D*—H⋯*A*	*D*—H	H⋯*A*	*D*⋯*A*	*D*—H⋯*A*
N3—H3*A*⋯Br1^i^	0.90 (5)	2.80 (4)	3.539 (4)	141 (5)

Symmetry code: (i) 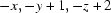.

**Fig. 1.**
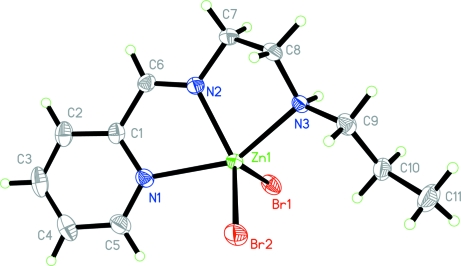
The molecular structure of (I) with ellipsoids drawn at the 30% probability level.

**Fig. 2.**
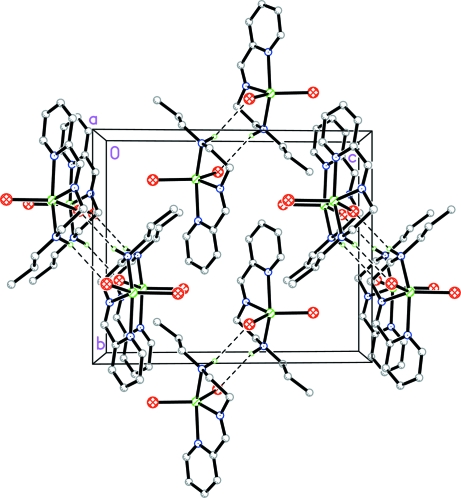
The crystal packing of (I), viewed along the a axis.

## supplementary crystallographic information

### Comment

Schiff bases have widely been used as versatile ligands in coordination chemistry (Biswas *et al.*, 2008; Wu *et al.*, 2008; Kawamoto *et al.*, 2008; Ali *et al.*, 2008; Habibi *et al.*, 2007), and their metal complexes are of great interest in many fields (Chen *et al.*, 2008; Yuan *et al.*, 2007; Tomat *et al.*, 2007; Darensbourg & Frantz, 2007). Zinc(II) is an important element in biological systems, functions as the active site of hydrolytic enzymes, such as carboxypeptidase and carbonic anhydrase where it is in a hard-donor coordination environment of nitrogen and oxygen ligands (Lipscomb & Sträter, 1996). In this paper, a new zinc(II) complex, (I), Fig. 1, with the Schiff base ligand *N*-propyl-*N*'-(1-pyridin-2-ylmethylidene)ethane-1,2-diamine has been synthesized and structurally characterized.

The Zn^II^ atom in (I) is five-coordinated by one imine N, one pyridine N, and one amine N atoms of the Schiff base ligand, and by two Br^-^ anions, in a distorted trigonal-bipyramidal coordination geometry. The coordinate bond lengths (Table 1) are typical and comparable to the corresponding values observed in similar zinc(II) Schiff base complexes (You & Zhu, 2006; Dapporto *et al.*, 2001). The bond angle N1—Zn1—N3 in the complex is 149.43 (15)° indicating a significant distortion from trigonal-bipyramidal coordination.

In the crystal structure, adjacent molecules are linked through intermolecular N–H···Br hydrogen bonds (Table 2), forming dimers (Fig. 2).

### Experimental

The Schiff base compound was prepared by the condensation of equimolar amounts of pyridine-2-carbaldehyde with *N*-propylethane-1,2-diamine in a methanol solution. The complex was prepared by the following method. To an anhydrous methanol solution (5 ml) of ZnBr_2_ (22.5 mg, 0.1 mmol) was added a methanol solution (10 ml) of the Schiff base compound (19.1 mg, 0.1 mmol) with stirring. The mixture was stirred for 30 min at room temperature and filtered. Upon keeping the filtrate in air for a few days, colorless block-shaped crystals formed at the bottom of the vessel on slow evaporation of the solvent.

### Refinement

H3A attached to N3 was located from a difference Fourier map and refined isotropically, with the N–H distance restrained to 0.90 (1) Å, and with *U*_iso_(H) fixed at 0.08 Å^2^. Other H atoms were placed in geometrically idealized positions and constrained to ride on their parent atoms, with C–H distances in the range 0.93–0.97Å and with *U*_iso_(H) = 1.2*U*_eq_(C) and 1.5*U*_eq_(methyl C).

### Crystal data

**Table:**

[ZnBr_2_(C_11_H_17_N_3_)]	*F*_000_ = 816
*M**_r_* = 416.47	*D*_x_ = 1.864 Mg m^−^^3^
Monoclinic, *P*2_1_/*n*	Mo *K*α radiation λ = 0.71073 Å
Hall symbol: -P 2yn	Cell parameters from 1994 reflections
*a* = 8.252 (4) Å	θ = 2.2–25.3º
*b* = 12.249 (5) Å	µ = 7.02 mm^−^^1^
*c* = 14.726 (6) Å	*T* = 298 (2) K
β = 94.562 (7)º	Block, colorless
*V* = 1483.8 (11) Å^3^	0.32 × 0.30 × 0.30 mm
*Z* = 4	

### Data collection

**Table:**

Bruker APEXII CCD area-detector diffractometer	3378 independent reflections
Radiation source: fine-focus sealed tube	2167 reflections with *I* > 2σ(*I*)
Monochromator: graphite	*R*_int_ = 0.083
*T* = 298(2) K	θ_max_ = 27.5º
ω scans	θ_min_ = 2.2º
Absorption correction: multi-scan(SADABS; Sheldrick, 2004)	*h* = −10→10
*T*_min_ = 0.212, *T*_max_ = 0.227	*k* = −15→15
12333 measured reflections	*l* = −18→19

### Refinement

**Table:**

Refinement on *F*^2^	Secondary atom site location: difference Fourier map
Least-squares matrix: full	Hydrogen site location: inferred from neighbouring sites
*R*[*F*^2^ > 2σ(*F*^2^)] = 0.045	H atoms treated by a mixture of independent and constrained refinement
*wR*(*F*^2^) = 0.105	*w* = 1/[σ^2^(*F*_o_^2^) + (0.0211*P*)^2^] where *P* = (*F*_o_^2^ + 2*F*_c_^2^)/3
*S* = 1.01	(Δ/σ)_max_ = 0.001
3378 reflections	Δρ_max_ = 0.91 e Å^−^^3^
159 parameters	Δρ_min_ = −0.80 e Å^−^^3^
1 restraint	Extinction correction: SHELXL, Fc^*^=kFc[1+0.001xFc^2^λ^3^/sin(2θ)]^-1/4^
Primary atom site location: structure-invariant direct methods	Extinction coefficient: 0.0140 (7)

### Special details

**Table:**

Geometry. All e.s.d.'s (except the e.s.d. in the dihedral angle between two l.s. planes) are estimated using the full covariance matrix. The cell e.s.d.'s are taken into account individually in the estimation of e.s.d.'s in distances, angles and torsion angles; correlations between e.s.d.'s in cell parameters are only used when they are defined by crystal symmetry. An approximate (isotropic) treatment of cell e.s.d.'s is used for estimating e.s.d.'s involving l.s. planes.
Refinement. Refinement of *F*^2^ against ALL reflections. The weighted *R*-factor *wR* and goodness of fit *S* are based on *F*^2^, conventional *R*-factors *R* are based on *F*, with *F* set to zero for negative *F*^2^. The threshold expression of *F*^2^ > σ(*F*^2^) is used only for calculating *R*-factors(gt) *etc*. and is not relevant to the choice of reflections for refinement. *R*-factors based on *F*^2^ are statistically about twice as large as those based on *F*, and *R*- factors based on ALL data will be even larger.

### Fractional atomic coordinates and isotropic or equivalent isotropic displacement parameters (Å^2^)

**Table:**

	*x*	*y*	*z*	*U*_iso_*/*U*_eq_	
Zn1	0.04170 (7)	0.30316 (4)	0.86078 (4)	0.02971 (19)	
Br1	−0.19476 (6)	0.34399 (5)	0.94074 (4)	0.0454 (2)	
Br2	0.00560 (8)	0.29983 (5)	0.69779 (4)	0.0524 (2)	
N1	0.0385 (5)	0.1154 (3)	0.8679 (3)	0.0359 (10)	
N2	0.2385 (5)	0.2563 (3)	0.9510 (3)	0.0338 (10)	
N3	0.1741 (5)	0.4581 (3)	0.8836 (3)	0.0296 (9)	
C1	0.1561 (6)	0.0745 (4)	0.9254 (3)	0.0351 (12)	
C2	0.1774 (8)	−0.0353 (4)	0.9398 (4)	0.0495 (15)	
H2	0.2599	−0.0611	0.9810	0.059*	
C3	0.0742 (9)	−0.1061 (5)	0.8922 (5)	0.0606 (18)	
H3	0.0849	−0.1811	0.9009	0.073*	
C4	−0.0449 (8)	−0.0652 (5)	0.8318 (5)	0.0575 (17)	
H4	−0.1150	−0.1117	0.7978	0.069*	
C5	−0.0591 (7)	0.0466 (5)	0.8222 (4)	0.0489 (15)	
H5	−0.1412	0.0743	0.7818	0.059*	
C6	0.2635 (6)	0.1575 (4)	0.9712 (4)	0.0367 (12)	
H6	0.3474	0.1373	1.0138	0.044*	
C7	0.3456 (6)	0.3442 (4)	0.9865 (4)	0.0396 (13)	
H7A	0.4553	0.3172	1.0003	0.048*	
H7B	0.3068	0.3745	1.0417	0.048*	
C8	0.3422 (6)	0.4295 (4)	0.9126 (4)	0.0375 (13)	
H8A	0.4004	0.4941	0.9352	0.045*	
H8B	0.3958	0.4016	0.8611	0.045*	
C9	0.1638 (6)	0.5399 (4)	0.8098 (4)	0.0400 (13)	
H9A	0.1939	0.5061	0.7540	0.048*	
H9B	0.2404	0.5984	0.8252	0.048*	
C10	−0.0046 (6)	0.5869 (4)	0.7943 (4)	0.0426 (14)	
H10A	−0.0825	0.5278	0.7857	0.051*	
H10B	−0.0298	0.6277	0.8478	0.051*	
C11	−0.0207 (7)	0.6607 (5)	0.7126 (4)	0.0641 (19)	
H11A	0.0663	0.7129	0.7167	0.096*	
H11B	−0.1230	0.6983	0.7108	0.096*	
H11C	−0.0158	0.6180	0.6582	0.096*	
H3A	0.130 (7)	0.491 (4)	0.930 (3)	0.080*	

### Atomic displacement parameters (Å^2^)

**Table:**

	*U*^11^	*U*^22^	*U*^33^	*U*^12^	*U*^13^	*U*^23^
Zn1	0.0321 (3)	0.0296 (3)	0.0266 (3)	0.0004 (2)	−0.0029 (2)	−0.0032 (3)
Br1	0.0399 (3)	0.0451 (4)	0.0526 (4)	−0.0043 (2)	0.0125 (3)	−0.0179 (3)
Br2	0.0811 (5)	0.0481 (4)	0.0263 (3)	0.0018 (3)	−0.0070 (3)	−0.0066 (3)
N1	0.043 (3)	0.033 (2)	0.031 (3)	−0.003 (2)	0.002 (2)	−0.001 (2)
N2	0.039 (3)	0.032 (2)	0.029 (2)	0.000 (2)	−0.003 (2)	0.001 (2)
N3	0.036 (2)	0.027 (2)	0.026 (2)	−0.0041 (17)	0.0009 (19)	−0.0019 (18)
C1	0.046 (3)	0.031 (3)	0.029 (3)	0.002 (2)	0.010 (2)	0.005 (2)
C2	0.065 (4)	0.035 (3)	0.050 (4)	0.007 (3)	0.013 (3)	0.009 (3)
C3	0.087 (5)	0.028 (3)	0.071 (5)	0.002 (3)	0.032 (4)	0.004 (3)
C4	0.070 (4)	0.037 (4)	0.067 (5)	−0.010 (3)	0.019 (4)	−0.010 (3)
C5	0.044 (3)	0.047 (4)	0.056 (4)	−0.006 (3)	0.000 (3)	−0.011 (3)
C6	0.041 (3)	0.038 (3)	0.030 (3)	0.008 (2)	−0.002 (2)	0.000 (2)
C7	0.042 (3)	0.040 (3)	0.035 (3)	−0.001 (2)	−0.011 (3)	−0.002 (3)
C8	0.037 (3)	0.033 (3)	0.041 (3)	−0.008 (2)	−0.002 (2)	−0.003 (3)
C9	0.046 (3)	0.033 (3)	0.041 (3)	−0.009 (2)	0.005 (3)	0.004 (3)
C10	0.044 (3)	0.032 (3)	0.052 (4)	0.002 (2)	0.003 (3)	0.007 (3)
C11	0.065 (4)	0.054 (4)	0.071 (5)	0.004 (3)	−0.004 (4)	0.030 (3)

### Geometric parameters (Å, °)

**Table:**

Zn1—N2	2.095 (4)	C4—C5	1.380 (8)
Zn1—N3	2.202 (4)	C4—H4	0.9300
Zn1—N1	2.303 (4)	C5—H5	0.9300
Zn1—Br2	2.3954 (13)	C6—H6	0.9300
Zn1—Br1	2.4102 (11)	C7—C8	1.508 (7)
N1—C5	1.313 (6)	C7—H7A	0.9700
N1—C1	1.334 (6)	C7—H7B	0.9700
N2—C6	1.259 (6)	C8—H8A	0.9700
N2—C7	1.463 (6)	C8—H8B	0.9700
N3—C8	1.461 (6)	C9—C10	1.505 (7)
N3—C9	1.477 (6)	C9—H9A	0.9700
N3—H3A	0.90 (5)	C9—H9B	0.9700
C1—C2	1.371 (7)	C10—C11	1.501 (7)
C1—C6	1.475 (7)	C10—H10A	0.9700
C2—C3	1.369 (8)	C10—H10B	0.9700
C2—H2	0.9300	C11—H11A	0.9600
C3—C4	1.367 (8)	C11—H11B	0.9600
C3—H3	0.9300	C11—H11C	0.9600
			
N2—Zn1—N3	77.83 (16)	N1—C5—H5	118.7
N2—Zn1—N1	73.04 (15)	C4—C5—H5	118.7
N3—Zn1—N1	149.43 (15)	N2—C6—C1	118.2 (5)
N2—Zn1—Br2	131.24 (11)	N2—C6—H6	120.9
N3—Zn1—Br2	100.86 (11)	C1—C6—H6	120.9
N1—Zn1—Br2	91.57 (11)	N2—C7—C8	106.0 (4)
N2—Zn1—Br1	111.33 (12)	N2—C7—H7A	110.5
N3—Zn1—Br1	99.08 (11)	C8—C7—H7A	110.5
N1—Zn1—Br1	99.94 (11)	N2—C7—H7B	110.5
Br2—Zn1—Br1	116.88 (4)	C8—C7—H7B	110.5
C5—N1—C1	118.1 (5)	H7A—C7—H7B	108.7
C5—N1—Zn1	128.8 (4)	N3—C8—C7	109.8 (4)
C1—N1—Zn1	113.1 (3)	N3—C8—H8A	109.7
C6—N2—C7	122.7 (4)	C7—C8—H8A	109.7
C6—N2—Zn1	121.2 (3)	N3—C8—H8B	109.7
C7—N2—Zn1	116.1 (3)	C7—C8—H8B	109.7
C8—N3—C9	112.0 (4)	H8A—C8—H8B	108.2
C8—N3—Zn1	106.6 (3)	N3—C9—C10	111.7 (4)
C9—N3—Zn1	118.3 (3)	N3—C9—H9A	109.3
C8—N3—H3A	109 (4)	C10—C9—H9A	109.3
C9—N3—H3A	105 (4)	N3—C9—H9B	109.3
Zn1—N3—H3A	106 (4)	C10—C9—H9B	109.3
N1—C1—C2	122.9 (5)	H9A—C9—H9B	107.9
N1—C1—C6	114.3 (4)	C11—C10—C9	111.9 (5)
C2—C1—C6	122.7 (5)	C11—C10—H10A	109.2
C3—C2—C1	118.4 (6)	C9—C10—H10A	109.2
C3—C2—H2	120.8	C11—C10—H10B	109.2
C1—C2—H2	120.8	C9—C10—H10B	109.2
C4—C3—C2	119.1 (6)	H10A—C10—H10B	107.9
C4—C3—H3	120.5	C10—C11—H11A	109.5
C2—C3—H3	120.5	C10—C11—H11B	109.5
C3—C4—C5	118.8 (6)	H11A—C11—H11B	109.5
C3—C4—H4	120.6	C10—C11—H11C	109.5
C5—C4—H4	120.6	H11A—C11—H11C	109.5
N1—C5—C4	122.7 (6)	H11B—C11—H11C	109.5

### Hydrogen-bond geometry (Å, °)

**Table:**

*D*—H···*A*	*D*—H	H···*A*	*D*···*A*	*D*—H···*A*
N3—H3A···Br1^i^	0.90 (5)	2.80 (4)	3.539 (4)	141 (5)

Symmetry codes: (i) −*x*, −*y*+1, −*z*+2.
